# Transcriptome Analysis Provides Insights into Water Immersion Promoting the Decocooning of *Osmia excavata* Alfken

**DOI:** 10.3390/insects15040288

**Published:** 2024-04-18

**Authors:** Guiping Wang, Guangzhao Wang, Jiale Li, Yixiang Ma, Yinwei You, Zizhang Zhou, Yunhe Zhao, Xingyuan Men, Yingying Song, Yi Yu

**Affiliations:** 1 Institute of Plant Protection, Shandong Academy of Agricultural Sciences, Jinan 250100, China; wangguiping1018@126.com (G.W.); wangguangzhao3675@163.com (G.W.); myx4362@163.com (Y.M.); yyw30000@163.com (Y.Y.); menxy2000@hotmail.com (X.M.); syysdau@126.com (Y.S.); 2 School of Agricultural Science and Technology, Shandong Academy of Agricultural Engineering, Jinan 251100, China; 15275823176@163.com; 3 School of Life Sciences, Yantai University, Yantai 264005, China; 4 College of Life Sciences, Shandong Agricultural University, Taian 271018, China; zhouzz@sdau.edu.cn (Z.Z.); yhzhao@sdau.edu.cn (Y.Z.)

**Keywords:** *Osmia excavata* Alfken, water immersion, decocooning, transcriptome sequencing, differentially expressed genes, KEGG pathways

## Abstract

**Simple Summary:**

*Osmia excavata* Alfken is an excellent pollinator. A variety of factors affect the rate of *O. excavata* release from its cocoon. However, rapid improvement of the concentrated decocooning of *O. excavata* has not been sufficiently investigated. In this study, we examined the effect of water immersion on the decocooning rate of *O. excavata*. Our results showed that water immersion significantly improved *O. excavata* decocooning. This will provide technical support to improve effective application of *O. excavata*. Illumina Novaseq™ 6000 transcriptome sequencing showed muscle-related functions play important roles in *O. excavata* decocooning in response to water immersion.

**Abstract:**

The timing of decocooning and nesting during the flowering period are crucial for the reproduction and pollination activities of *Osmia excavata*. In order to improve the pollination efficiency of *O*. *excavata*, it is crucial to find a way to break the cocoon quickly. Our results showed that the decocooning rates at 6, 12, 24, 36, 48, and 72 h after 30 min of water immersion (WI) were 28.67%, 37.33%, 37.33%, 41.33%, 44.33%, and 53.00%, respectively. The decocooning rate fold of 6 h was 14.33 compared with the control group. Transcriptome sequencing resulted in 273 differentially expressed genes (DEGs) being identified between the WI and control groups. Gene Ontology (GO) and Kyoto Encyclopedia of Genes and Genomes (KEGG) analysis showed that muscle-related functions play important roles in *O. excavata* decocooning in response to WI. Cluster analysis also showed that DEGs in cardiac muscle contraction and adrenergic signaling in cardiomyocytes were up-regulated in response to WI-promoted decocooning. In conclusion, the rate of decocooning can be improved by WI in a short time. During WI-promoted decocooning, muscle-related pathways play an important role. Therefore, the application of this technology will improve the pollination effect of *O*. *excavata*.

## 1. Introduction

The wild solitary bee *Osmia excavata* Alfken (Hymenoptera: Megachilidae) is an excellent pollinator that is widely distributed in the northern provinces of China [1]. As compared to other pollinating insects, the advantages of *O*. *excavata* include rapid, specific, and efficient pollination, tolerance to low temperatures, activity in the early spring, simple management, and low cost [2,3]. *Osmia excavata* has been used for more than 30 years to ensure the production, quality, and sustainability of various fruits and cruciferous vegetables in northern China [4,5,6].

Spinning and cocooning are instincts of many insects, providing a shelter to the residing pupae against adverse factors. After the metamorphosis of pupa into adult, the adult must break open the cocoon to emerge, which is called decocooning [7]. *O. excavata* builds nests with mud in holes under rocks and tiles and lives in the nests for about 320 days each year. It feeds on pollen (nectar) and exhibits generational breeding, where the offspring survive by feeding on pollen in the nest chamber [1,8]. *O. excavata* has one generation per year (egg, larva, pupa, and adult). The adult overwinters in the cocoon. The process of overwintering includes two stages, diapause and postdiapause, and the nutrient status in the body of the bee changes accordingly before and after diapause [9,10,11,12]. The time of releasing diapause is around late February. When the inside and outside temperature is stable above 12 °C, the dormant adult *O. excavata* wakes up, automatically breaks the cocoon, and moves out of the nest or visits flowers. In order to ensure the pollination effect of *O. excavata*, the cocoon should be refrigerated at 0–4 °C, so that its nesting activity can coincide with the flowering period of fruit trees, and so achieve pollination.

Fruit growers release *O. excavata* in orchards to improve the fruit setting rate. A study found that the release density, pollination distance, and selection of flowers on apple trees affected the pollination effect of the bee [13]. The timing of decocooning and nesting during the flowering period is crucial for reproduction of *O*. *excavata* [14]. The abundance of eggs produced by female *O. excavata* is closely related to the amount of collected pollen and nectar. When external sources of nectar are scarce, the female bees produce fewer pollen clusters and eggs [15]. The optimal time for pollination of pear trees is from day 1 to day 3 of the 7-day flowering period, as successful pollination significantly decreases after day 5 [16]. The full flowering stage of apple trees occurs 5–10 days after the inflorescence separation stage [17]. A sufficient number of pollinating insects is needed during the flowering period to ensure effective pollination. The timing of bee release varies by fruit tree species: for peach trees it is generally recommended that bees are released at about 20% bloom and for apple trees at 3–5% bloom. Under normal circumstances, after the bee cocoons are placed in the field, 7–10 d is sufficient for all cocoons to be broken [13,18].

A variety of factors affect the rate of *O*. *excavata* decocooning [19]. However, rapid improvement of concentrated *O*. *excavata* decocooning has not been sufficiently investigated. In order to improve *O*. *excavata* pollination efficiency, it is important to find a way to break the cocoon quickly. We found that water immersion (WI) for 30 min significantly improved *O*. *excavata* decocooning. This will provide technical support to improve effective application of *O*. *excavata.* Transcriptome sequencing resulted in 273 differentially expressed genes (DEGs) being identified between the WI and control groups. A total of 67 DEGs (24.54%) were up-regulated and 206 DEGs (75.46%) were down-regulated. Gene Ontology (GO) and Kyoto Encyclopedia of Genes and Genomes (KEGG) enrichment analysis showed muscle-related functions play important roles in *O. excavata* decocooning in response to WI.

## 2. Materials and Methods

### 2.1. Experimental Insects and Influence of WI on O. excavata Decocooning

In February 2023, *O. excavata* cocoons were purchased from Yantai Bifeng Agricultural Science and Technology Co., Yantai, China. The cocoons were kept at a low temperature (4 °C) in a refrigerator to prevent cocoon breaking before the flowering period of fruit trees. The experiment was carried out in March, when the bees were dormant. The *O. excavata* cocoons (n = 100 each) were exposed to WI for 15, 30, 60, or 120 min, respectively, to determine the optimal duration of WI to promote decocooning. After treatment, the cocoons were transferred to individual feeding boxes and the number of broken cocoons was counted at 6, 12, 24, 36, 48, and 72 h at 25 °C. The control group did not receive any treatment. The number of broken cocoons in the control group was also counted at 6, 12, 24, 36, 48, and 72 h at 25 °C. A total of 100 cocoons were used in each replicate. The experiment was independently repeated three times for each group.

### 2.2. Transcriptome Samples

According to the WI test above, the WI for 30 min was determined as the optimal duration to promote decocooning. Thus, the bees were separated into a WI-promoted decocooning group (WI group) or control group for the transcriptome experiments. In the WI group, the decocooned *O. excavata* were collected every 30 min and collected six times in total. That is, the decocooned *O. excavata* in the first 3 h were the experimental group (WI group). At 3 h, no bees in the control group broke from cocoons. Therefore, we took the *O. excavata* in the cocoon as the control group. The bees were collected under sterile conditions, surface disinfected with 75% alcohol, rinsed with sterilized water, dried, frozen in liquid nitrogen, and stored at −80 °C. Each sample contained three bees.

### 2.3. RNA Extraction, Library Construction, and Transcriptomic Sequencing

Total RNA was isolated and purified from six samples using TRIzol reagent (Invitrogen, Carlsbad, CA, USA). The RNA amount and purity of each sample was quantified using a NanoDrop ND-1000 (NanoDrop, Wilmington, DE, USA). Six high-quality RNA samples were used to construct the sequencing library. The poly (A) RNA was purified using Dynabeads Oligo (dT)25-61005 (Thermo Fisher, Waltham, CA, USA). A cDNA library was synthesized using RNA as a template. The size was 300 ± 50 bp. The RNA-seq analysis was performed using an Illumina Novaseq™ 6000 platform.

### 2.4. Differential Expression Analysis

Firstly, Cutadapt (1.9) [20] (https://cutadapt.readthedocs.io/en/stable/,version:cutadapt-1.9, accessed on 28 April 2023) was used to remove the unsatisfactory reads. FastQC (http://www.bioinformatics.babraham.ac.uk/projects/fastqc/, 0.10.1, accessed on 28 April 2023) was used to verify the clean data quality. De novo assembly of the transcriptome was performed with Trinity (2.15) [21]. It was used to cluster the assembled transcripts based on sequence similarity. The longest sequence among these similar transcripts was selected and labeled as a unigene. DIAMOND (2.0.15) was used to annotate all assembled unigenes with the non-redundant (Nr) protein (http://www.ncbi.nlm.nih.gov/, accessed on 28 April 2023), Gene Ontology (GO) (http://www.geneontology.org, accessed on 28 April 2023), SwissProt (http://www.expasy.ch/sprot/, accessed on 28 April 2023), Kyoto Encyclopedia of Genes and Genomes (KEGG) (http://www.kegg.jp/kegg/, accessed on 28 April 2023), and eggNOG (http://eggnogdb.embl.de/, accessed on 28 April 2023) databases and Pfam (http://pfam.xfam.org/, accessed on 28 April 2023) [22]. Salmon (1.9.0) [23] was used to quantify unigenes using transcripts per kilobase of exon model per million mapped reads (TPM) [24]. The differentially expressed unigenes were selected with log_2_ (fold change) > 1 or log_2_ (fold change) < −1 and with false discovery rate (FDR) < 0.05 using R package edgeR (3.40.2) [25].

### 2.5. Sample Correlation Analysis

To evaluate the trend of inter-group separation and intra-group aggregation of the samples, principal component analysis (PCA) was performed using the princomp function in R.

### 2.6. GO and KEGG Enrichment Analysis

The GO and KEGG enrichment analyses of DEGs were performed in the GO and KEGG databases, respectively. Firstly, the number of genes with significant differences in a specific GO term or KEGG pathway (S), total number of significant differential genes in a GO term or KEGG pathway (TS), the number of genes annotated as a specific GO term or KEGG pathway (B), and total background gene number (TB) for each GO term or KEGG pathway were calculated. Then, the hypergeometric distribution was used to find the GO term or KEGG pathway that is significantly enriched in DEGs. Corrected *p*-values < 0.05 are considered significantly enriched.

### 2.7. Statistical Analysis

In this study, data are presented as mean ± standard error of the mean (SEM) of the three technical replicates. GraphPad Prism 6.01 Software (GraphPad Software Inc., San Diego, CA, USA) was used for data analysis. Statistical analyses were determined by the two-tailed, unpaired Student’s *t*-test, with *p* < 0.05 considered significant.

## 3. Results

### 3.1. Influence of WI on Decocooning

**Figure 1:**
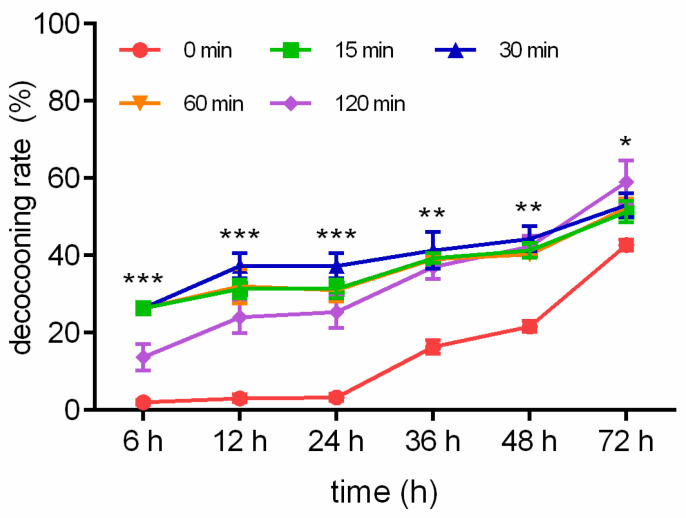
Different WI-promoted decocooning rates of *O. excavata* at 6, 12, 24, 36, 48, and 72 h. 0 min: control group; 15 min: WI for 15 min; 30 min: WI for 30 min; 60 min: WI for 60 min; 120 min: WI for 120 min. The difference between the control group and WI for 30 min was analyzed. Data are expressed as mean ± SEM; n = 3; * *p* < 0.05, ** *p* < 0.01, and *** *p* < 0.001 vs. the control group.

The decocooning rates in response to WI are described in Figure 1. The decocooning rates when exposed to WI for 15 min were 26.33%, 31.33%, 31.33%, 39.33%, 41.33%, and 51.33% after 6, 12, 24, 36, 48, and 72 h, respectively. The decocooning rates when exposed to WI for 30 min were 28.67%, 37.33%, 37.33%, 41.33%, 44.33%, and 53.00% after 6, 12, 24, 36, 48, and 72 h, respectively. The decocooning rates when exposed to WI for 60 min were 23.67%, 32.00%, 31.00%, 39.00%, 40.33%, and 52.33% after 6, 12, 24, 36, 48, and 72 h, respectively. The decocooning rates when exposed to WI for 120 min were 13.67%, 24.00%, 25.33%, 37.00%, 42.33%, and 59.00% after 6, 12, 24, 36, 48, and 72 h, respectively. The decocooning rates of the control group were 2.00%, 3.00%, 3.33%, 16.33%, 21.67%, and 42.67% after 6, 12, 24, 36, 48, and 72 h, respectively. Because there was overlap in the line chart of decocooning rate in the WI group, we chose WI for 30 min to compare with the control group. The decocooning rates when exposed to WI for 30 min at 6 (*p* < 0.001), 12 (*p* < 0.001), 24 (*p* < 0.001), 36 (*p* < 0.01), 48 (*p* < 0.01), and 72 h (*p* < 0.05) were significantly increased from that of the control group.

The fold changes to the decocooning rate in response to WI are described in Supplementary Figure S1. The relative fold changes to the decocooning rate of the groups exposed to WI for 15, 30, 60, and 120 min were increased by 13.17-, 14.33-, 11.83-, and 6.83-fold at 6 h after WI, respectively (Figure S1A); 10.44-, 12.44-, 10.67-, and 8.00-fold at 12 h after WI, respectively (Figure S1B); 9.40-, 11.20-, 9.80-, and 7.60-fold at 24 h after WI, respectively (Figure S1C); 2.41-, 2.53-, 2.39-, and 2.27-fold at 36 h after WI, respectively (Figure S1D); 1.91-, 1.07-, 0.91-, and 1.05-fold at 48 h after WI, respectively (Figure S1E); and 1.20-, 1.24-, 1.23-, and 1.38-fold at 72 h after WI, respectively (Figure S1F). Compared to the control group, WI for 30 min resulted in the best decocooning rate.

### 3.2. High-Throughput Sequencing Results

Three control samples and three WI samples were used to construct cDNA libraries and sequence the transcripts with an Illumina Novaseq™ 6000 platform. The raw reads ranged from 35,763,154 to 42,151,520, while raw bases were between 5.36 G and 6.32 G (Supplementary Table S1). The results of high-throughput sequencing identified 35,035,328, 38,226,266, and 40,595,706 valid reads in the control group and 38,569,092, 38,426,182, and 36,736,678 in the WI group. The Q20% and Q30 scores of all samples were >97.52% and >92.53%, respectively. The GC content was 41.06%–44.65%. The transcriptome data indicated that the quality of sequencing data was sufficient for subsequent analysis.

### 3.3. Principal Component Analysis (PCA)

Principal component analysis (PCA) reflected the difference of samples between groups and variability of samples in the same group. The results showed that samples were scattered between the control group and WI group (Supplementary Figure S2). There were differences between the control group and WI group. In addition, PCl and PC2 were responsible for 47.25% and 23.12% of the variation, respectively. This verified the stability and reliability of the experimental data. Interestingly, samples from the WI group were more homogeneous than those from the control group. This was similar to the study by Chen et al. [26].

### 3.4. DEGs in Response to WI

**Figure 2:**
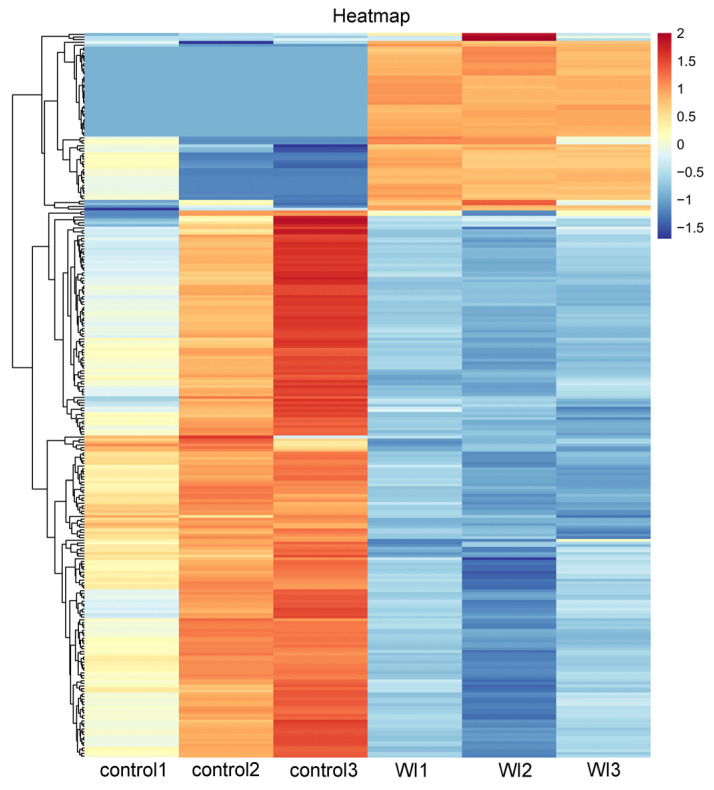
Hierarchical clustering heat map of DEGs between the WI and control groups. The horizontal coordinates represent the six samples and the clustering results of six samples. The vertical coordinates represent the 273 DEGs and the clustering results of all DEGs. The colors indicate the expression levels. High levels of expression are indicated in red and low expression is indicated in blue.

**Figure 3:**
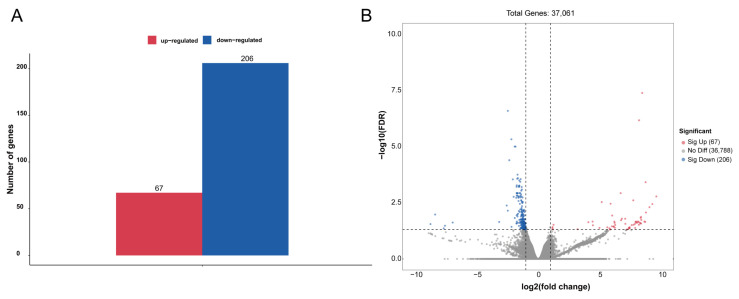
Analysis of DEGs between the WI and control groups. (**A**) The numbers of up-regulated (red column) and down-regulated (blue column) DEGs are summarized. (**B**) The volcano plot includes all DEGs. Up-regulated DEGs are indicated by red dots and down-regulated DEGs by blue dots. Non-DEGs are indicated by gray dots. The log_2_(fold change) values are shown on the *x* axis and −log_10_(FDR) values along the *y* axis. Genes with adjusted *p*-value ≤ 0.05 were considered differentially expressed.

To identify genes that displayed significant expression changes in response to WI-promoted decocooning, DEGs were analyzed. A hierarchical clustering heat map indicated that the DEGs in each group were similar, and the differences between groups were large. The DEG profiles were highly divergent between the WI and control groups (Figure 2). In total, 273 DEGs were identified (Figure 3 and Supplementary Table S2). More genes were down-regulated than up-regulated in response to WI. A total of 67 DEGs (24.54%) were up-regulated and 206 DEGs (75.46%) were down-regulated.

### 3.5. GO Enrichment Analysis Results of DEGs

**Figure 4:**
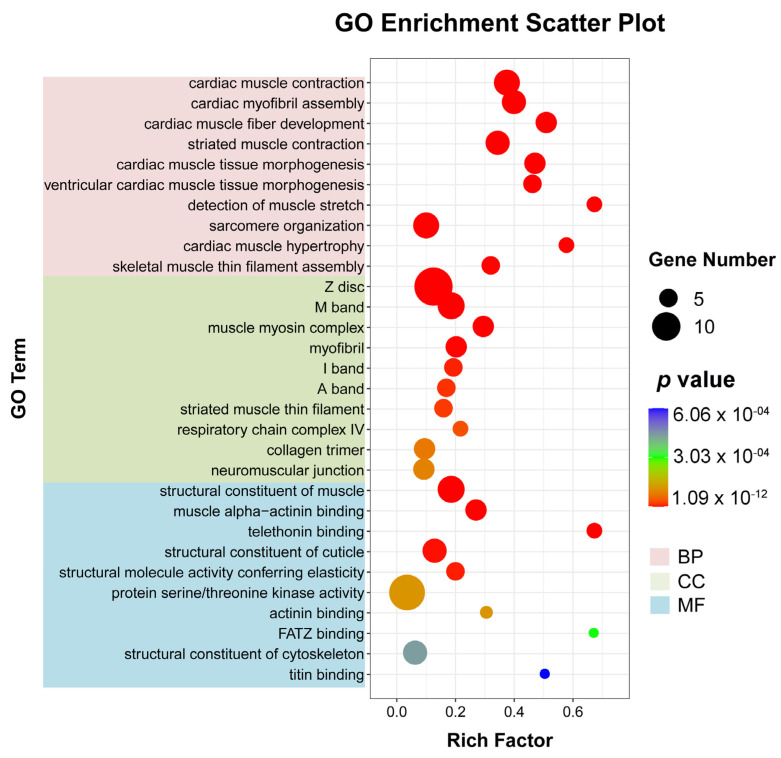
Bubble diagram of GO enrichment analysis of DEGs. The horizontal axis represents the enrichment degree (rich factor) and the vertical axis represents the enriched GO term. The pink section is BP; green is CC; blue is MF. Dot colors indicate different *p*-values. The smaller the *p*-value, the closer the color is to red. Rich factor represents the number of DEGs belonging to a GO term. The size of the dots (black dot) indicates the number of DEGs on the right.

To better understand the mechanism of WI-promoted *O. excavata* decocooning, we further studied the pathways with significant differences in gene expression (adjusted *p*-value < 0.05) between the WI and control groups. Firstly, we used ggplot2 to display the GO enrichment analysis on all DEGs (Supplementary Table S3). There were 242 GO entries involved in significant enrichment of biological processes (BP), 45 in cellular components (CC), and 81 in molecular functions (MF) (Supplementary Table S3). The top 10 GO items enriched by the three processes were respectively selected for display (Figure 4 and Supplementary Table S3). In BP, cardiac muscle contraction (nine, *p* = 1.0932 × 10^−12^), cardiac myofibril assembly (seven, *p* = 2.8860 × 10^−10^), cardiac muscle fiber development (six, *p* = 8.8860 × 10^−10^), striated muscle contraction (seven, *p* = 1.0276 × 10^−9^), and cardiac muscle tissue morphogenesis (six, *p* = 1.6363 × 10^−9^) were significantly enriched. In CC, Z disc (fourteen, *p* = 7.8804 × 10^−12^), M band (eight, *p* = 1.8327 × 10^−8^), muscle myosin complex (six, *p* = 4.8355 × 10^−8^), myofibril (six, *p* = 6.0290 × 10^−7^), and I band (five, *p* = 6.8789 × 10^−6^) were significantly enriched. In MF, structural constituent of muscle (eight, *p* = 1.8327 × 10^−8^), muscle alpha-actinin binding (six, *p* = 8.8445 × 10^−8^), telethonin binding (four, *p* = 1.5166 × 10^−7^), structural constituent of cuticle (seven, *p* = 2.1754 × 10^−6^), and structural molecule activity conferring elasticity (five, *p* = 5.6514 × 10^−6^) were significantly enriched. These showed that a large number of muscle-related DEGs were in the 30 GO terms that were most significantly enriched in the enrichment analysis results.

### 3.6. KEGG Pathways Associated with the DEGs

**Figure 5:**
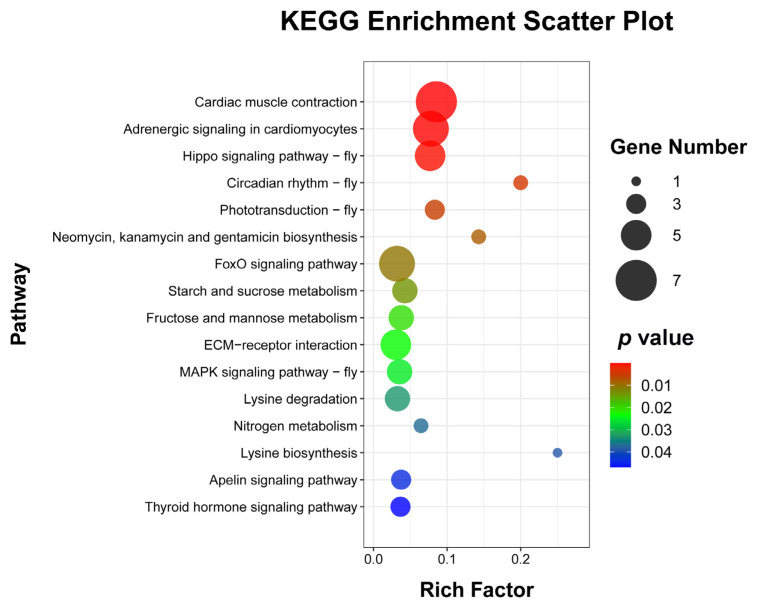
Bubble diagram of enriched KEGG pathways of DEGs. The abscissa represents the ratio of the DEGs to all the genes annotated in a particular pathway. The ordinate represents the enriched KEGG pathway. The number of DEGs contained in each pathway is represented by the size of the dots. The size of the *p*-value is indicated by the color of the dot. Blue is the highest and red is the lowest. Gene number (black dot) and *p*-value are shown on the right.

**Figure 6:**
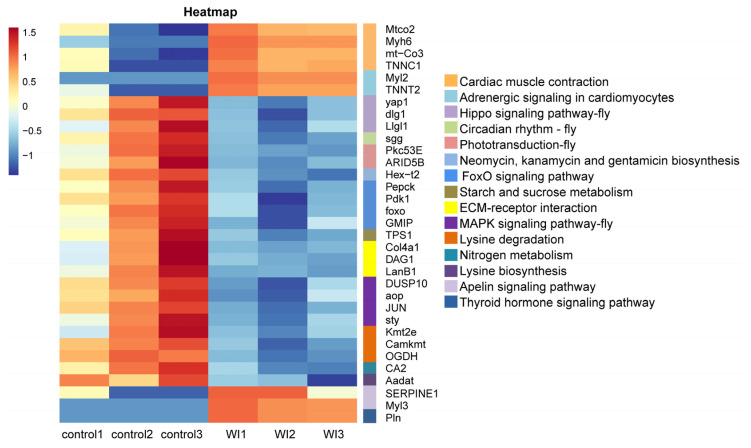
Cluster analyses of DEGs in 16 enriched KEGG pathways. The horizontal axis represents the samples and sample clustering results. The vertical axis represents the DEGs in 16 KEGG enrichment pathways and the clustering results of these DEGs. The 16 enriched KEGG pathways are shown on the right. Different colors indicate the different expression levels of DEGs. Red represents high expression, blue represents low expression.

To explore the enrichment pathways of DEGs, KEGG enrichment analysis of DEGs was performed. The DEGs were significantly enriched in 16 KEGG pathways, including cardiac muscle contraction (seven, *p* = 1.5024 × 10^−5^), adrenergic signaling in cardiomyocytes (six, *p* = 0.0001), Hippo signaling pathway—fly (five, *p* = 0.0004), circadian rhythm—fly (two, *p* = 0.0041), phototransduction—fly (three, *p* = 0.0052), neomycin, kanamycin, and gentamicin biosynthesis (two, *p* = 0.0081), FoxO signaling pathway (six, *p* = 0.0105), starch and sucrose metabolism (four, *p* = 0.0137), fructose and mannose metabolism (four, *p* = 0.0204), ECM–receptor interaction (five, *p* = 0.0233), MAPK signaling pathway—fly (four, *p* = 0.0252), lysine degradation (four, *p* = 0.0330), nitrogen metabolism (two, *p* = 0.0371), lysine biosynthesis (one, *p* =0.0388), apelin signaling pathway (three, *p* = 0.0441), and thyroid hormone signaling pathway (three, *p* = 0.0469) (Figure 5 and Supplementary Table S4). Cluster analysis was performed on the DEGs in 16 significantly enriched KEGG pathways (Figure 6). The hierarchical clustering heat map showed that DEGs in cardiac muscle contraction, adrenergic signaling in cardiomyocytes, apelin signaling pathway, and thyroid hormone signaling pathway were up-regulated in response to WI-promoted *O. excavata* decocooning. However, the DEGs in the Hippo signaling pathway, FoxO signaling pathway, MAPK signaling pathway, and other pathways were down-regulated.

## 4. Discussion

It is very important for the pollination effect of *O. excavata* for the time of decocooning and flowering of plants to coincide [14]. A variety of factors affect the *O. excavata* decocooning rate [19]. (1) When the weather is clear and temperature is high, the cocoon emergence speed is fast, and vice versa it is slow. When the late storage temperature was high, the decocooning was rapid. (2) The water content of the cocoon shell is related to the decocooning speed. After storage in winter and spring, the cocoon shell loses water and becomes harder. The rate of cocoon breaking slows and *O. excavata* can even die. (3) Low-temperature storage time affects cocoon emergence of adult bees. If the cocoon is stored at a low temperature for a long time, the adult bee can adapt to a low temperature by continuing diapause, but it cannot escape from the cocoon for a long time to supplement its nutrition and so will die of hunger. So, finding a way for *O. excavata* to quickly emerge from cocoons is crucial. In this study, WI for 30 min dramatically promoted *O. excavata* decocooning (Figure 1 and Supplementary Figure S1). WI can rapidly increase the rate of decocooning in a short period of time, so as to better pollinate fruit trees at the peak of flowering. Therefore, this technique can be applied to improve the pollination efficiency of *O. excavata* and increase fruit production.

In this study, PCA data showed heterogeneity in the control group (Supplementary Figure S2). This may be related to the life history of *O. excavata*. *O. excavata* is a solitary pollinator that breeds one generation per year. There are four developmental stages in the life history of *O. excavata*: egg (3–4 days), larva (20–25 days), pupa (25–30 days), and adult [27]. Under normal circumstances, differences in the developmental period lead to developmental differences between individuals. Therefore, this may cause the heterogeneity of the control group samples. Because of this reason, *O. excavata* undergoes gradual automatic decocooning at the peak of flowering, and the time of decocooning is not concentrated. In the production practice of applying *Osmia* pollination, in order to improve the rate of decocooning, artificial decocooning is used. However, in the WI group, the *O. excavata* needed a rapid stress response in order to escape this stress. The physiological status of the samples in the WI group was more consistent, so the uniformity was better. Although there was heterogeneity in the control group, the cluster heat map showed that the DEGs were better clustered in the control group and WI group (Figure 2). In addition, the clustering difference of individual genes did not affect the overall clustering effect.

To further explore the mechanism of WI promoting *O. excavata* decocooning, we conducted GO and KEGG enrichment analysis of DEGs. The GO enrichment analysis showed that, compared with the control group, genes related to muscle-related functions were enriched, such as cardiac muscle contraction, cardiac myofibril assembly, cardiac muscle fiber development, striated muscle contraction, and cardiac muscle tissue morphogenesis (Figure 4 and Supplementary Table S3). The KEGG pathway analysis revealed significant changes in cardiac muscle contraction and adrenergic signaling in cardiomyocytes (Figure 5 and Supplementary Table S4). The DEGs in cardiac muscle contraction (*Mtco2*, *Myh6*, *mt-Co3*, and *TNNC1*) and in adrenergic signaling in cardiomyocytes (*Myl2* and *TNNT2*) were up-regulated in response to WI-promoted decocooning (Figure 6). These suggest that muscle-related function pathways play an important role in the regulation of WI promoting *O. excavata* decocooning. In addition, heat shock 70 kDa protein 1A (*HspA1A*) and heat shock protein beta-7 (*Hspb7*) were up-regulated in response to WI-promoted decocooning. Heat shock genes, or stress genes, code for a number of proteins that collectively form a stress defense system to resist various adverse factors. Heat shock genes are up-regulated in response common stress factors, such as higher temperatures, hypoxia, heavy metals, and others [28]. This indicated that WI was a stress for the *O. excavate* cocoon.

*O. excavata* forms a cocoon, pupates, emerges as an adult from the cocoon, and then enters diapause and dormancy. The outer layer of the cocoon is rough and the inner layer is fine and semi-permeable. The adult bee can exchange gas with the outside world through this semi-permeable membrane. When the cocoon is soaked in water, the water infiltrates the cocoon, although it cannot penetrate the membrane, it can hinder the gas exchange between the inside and the outside of the cocoon, resulting in hypoxia stress in the cocoon. So, we speculate that the WI treatment affects the oxygen (O_2_) and carbon dioxide (CO_2_) concentrations in the cocoon, resulting in a decrease in O_2_ and an increase in CO_2_. CO_2_ is a ubiquitous sensory cue that affects the behavior, physiology, metabolism, and survival of insects [29,30]. For example, the increase in CO_2_ concentration in the nests of honey bees and bumble bees causes their fanning activity [31,32]; CO_2_ treatment induces postdiapause in bumble bees [33]; in *Drosopbila melanogaster*, CO_2_ cues are thought to play a role in selecting food sources [34]. The larvae of cowpea bruchids respond to low oxygen by coordinating reduced energy requirements, strengthening cellular structure and muscle contraction [35]. Therefore, we hypothesize that the muscle-related pathways are the executive pathways for decocooning in *O. excavata* to escape from high CO_2_ stress. The CO_2_-sensing pathway may be the upstream pathway in which WI promotes decocooning. However, no significant enrichment of hypoxia-related pathways was found in our study. Further studies are needed to investigate this.

## 5. Conclusions

Our results showed that WI can rapidly increase the *O. excavata* decocooning rate in a short period of time. This will provide technical support to improve effective application of *O*. *excavata.* The GO and KEGG enrichment analysis showed that muscle-related functions play important roles in *O. excavata* decocooning in response to WI. However, further studies are needed to investigate this potential relationship.

## Data Availability

The raw data have been uploaded to the NCBI website (https://www.ncbi.nlm.nih.gov/bioproject/PRJNA1079670, accessed on 11 April 2024) using the BioProject ID PRJNA1079670.

## Supplementary Materials

The following supporting information can be downloaded at: https://www.mdpi.com/article/10.3390/insects15040288/s1, Figure S1: The relative fold changes to the decocooning rate of the groups exposed to WI for 15, 30, 60, and 120 min at 6 (A), 12 (B), 24 (C), 36 (D), 48 (E), and 72 h (F). Figure S2: Principal component analysis (PCA) of all 6 samples from WI and control group. Table S1: Quality control of transcriptome sequences. Table S2: Table of DEGs. Table S3: GO enrichment analysis. Sheet1: biological process; Sheet2: cellular component; Sheet3: molecular_function. Table S4: KEGG enrichment analysis.
