# Supplementary material for: Transcriptome Analysis Provides Insights into Water Immersion Promoting the Decocooning of Osmia excavata Alfken
Source: Insects. 2024 Apr 18;15(4):288. doi: 10.3390/insects15040288 (PMC11049900; doi:10.3390/insects15040288)
Supplement: Supplementary file 1 [file insects-15-00288-s001.zip › Supplementary Files-proofreading/Figure S1.pdf]

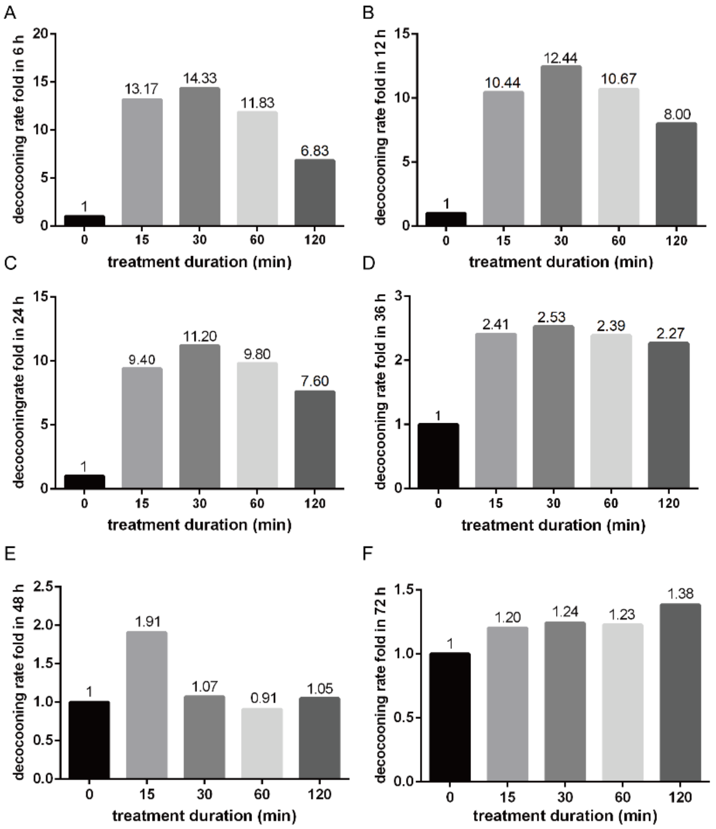

Figure S1. The relative fold changes to the decocooning rate of the groups exposed to WI for 15, 30, 60, and 120 min at 6 (A), 12 (B), 24 (C), 36 (D), 48 (E), and 72 h (F). 0: control group; 15: WI for 15 min; 30: WI for 30 min; 60: WI for 60 min; 120: WI for 120 min. The decocooning rate fold of the control group was set to 1.
