# Supplementary material for: Transcriptome Analysis Provides Insights into Water Immersion Promoting the Decocooning of Osmia excavata Alfken
Source: Insects. 2024 Apr 18;15(4):288. doi: 10.3390/insects15040288 (PMC11049900; doi:10.3390/insects15040288)
Supplement: Supplementary file 1 [file insects-15-00288-s001.zip › Supplementary Files-proofreading/Figure S2.pdf]

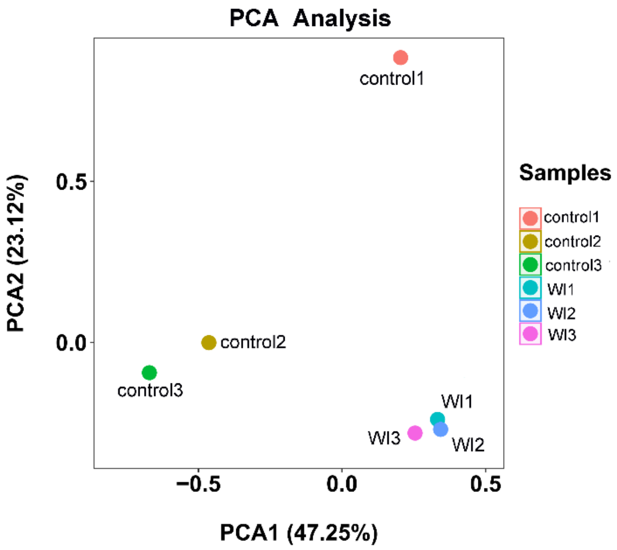

Figure S2. Principal component analysis (PCA) of all 6 samples from WI and control group. control1-control3: three replicates of the control group; WI1-WI3: three replicates of the WI group.
