# Supplementary material for: Transcriptome Analysis Provides Insights into Water Immersion Promoting the Decocooning of Osmia excavata Alfken
Source: Insects. 2024 Apr 18;15(4):288. doi: 10.3390/insects15040288 (PMC11049900; doi:10.3390/insects15040288)
Supplement: Supplementary file 1 [file insects-15-00288-s001.zip › Supplementary Files-proofreading/Table S1.pdf]

Table S1. Quality control of transcriptome sequences.

Sample	Raw Reads	Raw Bases (G)	Valid Reads	Valid Bases (G)	Valid (%)	Q20%	Q30%	GC%
control1	35763154	5.36	35035328	5.17	97.96	97.52	92.53	41.67
control2	39047244	5.86	38226266	5.65	97.90	97.67	93.05	42.57
control3	42151520	6.32	40595706	6.00	96.31	97.72	93.16	44.65
WI1	39582474	5.94	38569092	5.69	97.44	97.64	92.75	41.30
WI2	39605364	5.94	38426182	5.67	97.02	97.73	92.97	41.06
WI3	37972000	5.70	36736678	5.42	96.75	97.72	92.97	41.51

Note: Sample, sample name; Raw Reads, the number of reads in the offline data; Raw Bases, number of data bases to be dismounted; Valid Reads, number of valid reads; Valid Bases, number of bases of the valid data; Valid%, proportion of valid reads; Q20%, proportion of bases with mass values ≥ 20 (sequencing error rate < 0.01); Q30%, proportion of bases with mass values ≥ 30 (sequencing error rate < 0.001); GC%, GC content; control, control groups; WI, WI groups.
